# A graph-based machine learning framework identifies critical properties of FVIII that lead to hemophilia A

**DOI:** 10.3389/fbinf.2023.1152039

**Published:** 2023-05-10

**Authors:** Marcos V. Ferreira, Tatiane Nogueira, Ricardo A. Rios, Tiago J. S. Lopes

**Affiliations:** ^1^ Institute of Computing, Federal University of Bahia, Salvador, Brazil; ^2^ Center for Regenerative Medicine, National Center for Child Health and Development Research Institute, Tokyo, Japan

**Keywords:** protein structure, machine learning, bioinformatics, residue network, FVII, FVIIIa, graph neural network

## Abstract

**Introduction:** Blood coagulation is an essential process to cease bleeding in humans and other species. This mechanism is characterized by a molecular cascade of more than a dozen components activated after an injury to a blood vessel. In this process, the coagulation factor VIII (FVIII) is a master regulator, enhancing the activity of other components by thousands of times. In this sense, it is unsurprising that even single amino acid substitutions result in hemophilia A (HA)—a disease marked by uncontrolled bleeding and that leaves patients at permanent risk of hemorrhagic complications.

**Methods:** Despite recent advances in the diagnosis and treatment of HA, the precise role of each residue of the FVIII protein remains unclear. In this study, we developed a graph-based machine learning framework that explores in detail the network formed by the residues of the FVIII protein, where each residue is a node, and two nodes are connected if they are in close proximity on the FVIII 3D structure.

**Results:** Using this system, we identified the properties that lead to severe and mild forms of the disease. Finally, in an effort to advance the development of novel recombinant therapeutic FVIII proteins, we adapted our framework to predict the activity and expression of more than 300 *in vitro* alanine mutations, once more observing a close agreement between the *in silico* and the *in vitro* results.

**Discussion:** Together, the results derived from this study demonstrate how graph-based classifiers can leverage the diagnostic and treatment of a rare disease.

## 1 Introduction

Blood coagulation is a vital process that stops the bleeding that ensues after a blood vessel is damaged. Injuries to the endothelial cell layer of blood vessels lead to the production of Tissue Factor Pathway Inhibitor (TFPI), which in turn starts a cascade of signals that activate and inhibit more than a dozen factors and lead to the assembly of a fibrin clot at the site of injury (Hoffbrand et al., 2016). Evidently, any mutations to the genes involved in this delicate system lead to the disruption of this essential process; for instance, patients harboring mutations on the SERPINC1 gene are prone to develop thrombosis [the excessive formation of blood clots (Hoffbrand et al., 2016)]. On the other hand, inherited or spontaneous mutations to the Coagulation factor 8 (F8) lead to hemophilia A (HA), a coagulation disorder that cause patients to have uncontrolled bleeding episodes.

This is an X-linked heritable disease affecting approximately 1 in every 5,000–10,000 live male births (Hoffbrand et al., 2016), and as a result, the blood coagulation cascade is impaired to different extents depending on the type of mutation on the F8 gene. Disease symptoms may vary from mild (clotting activity level 5%–40%, with only rare bleeding episodes), to moderate (clotting activity 1%–5%, more frequent episodes), and severe [clotting activity < 1%, permanent bleeding risk and chronic joint damage (Lee et al., 2014)].

Although it is a relatively rare disorder, the coagulation pathway is well-characterized and treatment options are improving since the 1950s, evolving from blood-derived FVIII concentrates (Lee et al., 2014) to recombinant proteins (Peters and Harris, 2018), monoclonal antibodies (Kitazawa et al., 2012; Østergaard et al., 2021) and gene therapy (Nathwani, 2019). However, current treatment options still have major issues that have to be addressed (Lenting et al., 2017), for instance, it is of paramount importance to improve the half-life of recombinant FVIII proteins (currently ∼ 12–19 h), as well as its immunogenic profile to avoid the development of neutralizing antibodies, a condition affecting 30% of patients (Peters and Harris, 2018).

To this end, a deep understanding of the FVIII protein structure is essential. Using genetic information and protein structure properties, previous studies started to uncover aspects of single amino acid changes and their relation to severe or mild forms of HA (Doss, 2012). However, the lack of strict data curation and the lack of advanced statistical and machine learning methods hampered the mechanistic understanding and prediction of the effect of novel mutations on the FVIII protein.

In this study, to predict the degree of dysfunction that mutations cause in this protein, we used a graph representation of the FVIII that we established previously (Lopes et al., 2021a), and the mutation profile of 5,793 patients diagnosed with HA. We used this information and other structural and evolutionary properties of FVIII as input to 4 different graph-based neural network architectures (GNN), and found that this setup is highly efficient to learn the underlying properties of the FVIII architecture, and predict with good accuracy the effect of single-point non-synonymous mutations.

Moreover, aiming at creating recombinant FVIII proteins with improved half-life, immunogenic and folding profiles (Prezotti et al., 2022), we retrained these models to predict the coagulation activity of more than 300 alanine mutations. As a result, we found that the GNN models reliably predict the reduction in the activity of FVIII, effectively emulating *in silico* the results of costly and laborious *in vitro* assays.

In summary, this study builds on our previous efforts and demonstrates the feasibility of using GNNs to advance the understanding of a rare disease. We named this framework GNN-HemA and made it open-source, anticipating that the community will reproduce our findings and extend it to study diseases beyond hemophilia.

## 2 Results

### 2.1 Creation of the FVIII residue network

The FVIII protein has 2,332 amino acids and is composed of 5 domains (A1-A2-B-A3-C1-C2) (Childers et al., 2022). It circulates bound to the von Willebrand Factor (vWF), and after being activated via thrombin-mediated cleavage of some residues, it becomes detached from vWF, loses its B domain and changes into its activated form (FVIIIa) (Childers et al., 2022). As co-factor for the coagulation factor IXa, FVIIIa binds to the phospholipid membrane of activated platelets and enhances its activity more than 100,000 times (Lee et al., 2014). Together, they form the so-called tenase complex to activate the coagulation factor X (FX) into FXa. In turn, FXa converts prothrombin to thrombin, already close to the end of the coagulation cascade [i.e., the formation of a stable fibrin clot (Lee et al., 2014)].

**FIGURE 1:**
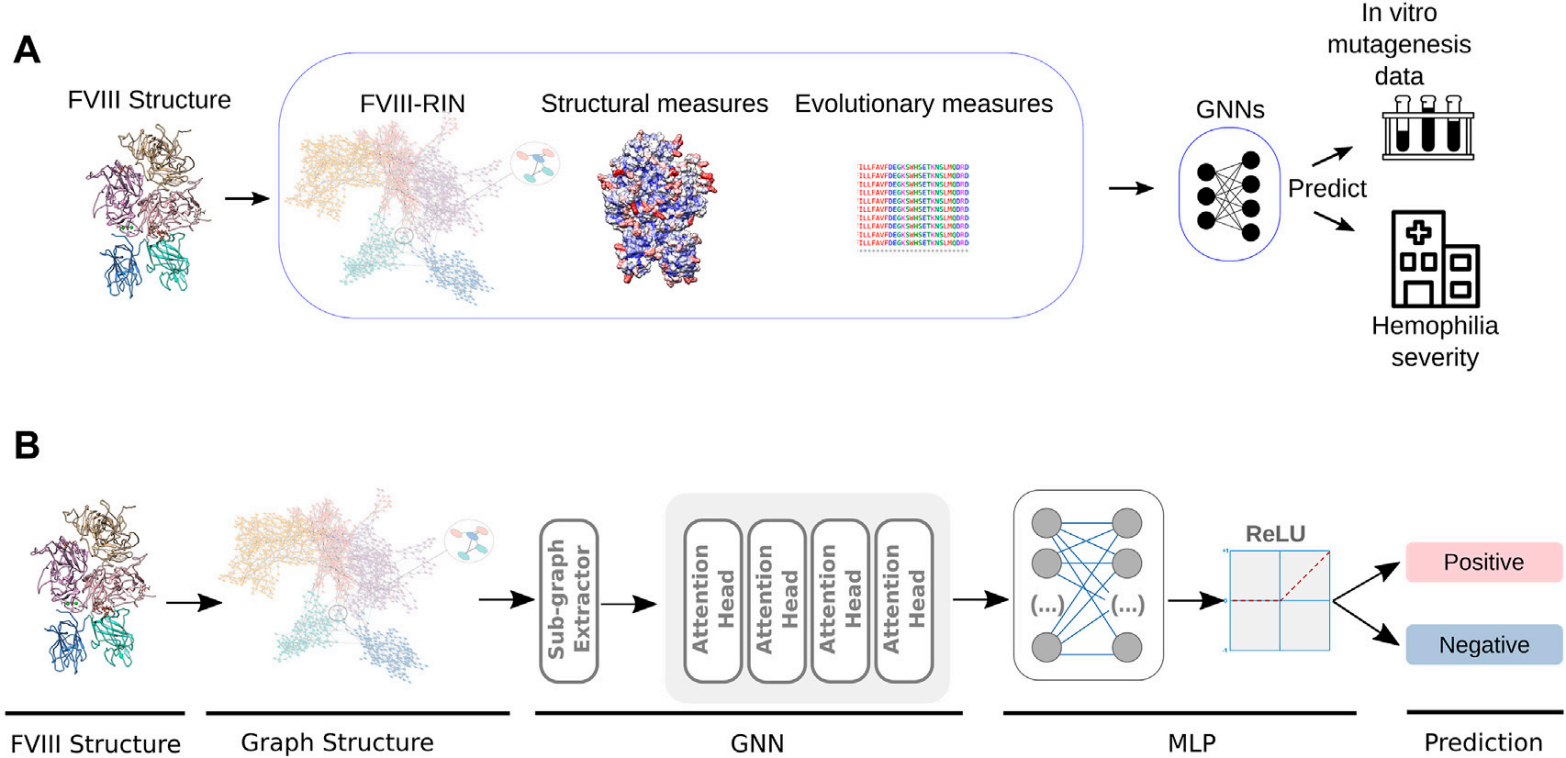
Design of the GNN-HemA. **(A)** From the pre-processed FVIII structure, we generated a residue network, obtained structural measures like solvent accessible area as well as a conservation score for each residue. This served as input for GNN classifiers, that were trained to predict the severity of 626 patients with HA, as well as the coagulation activity of more than 300 alanine mutant FVIII constructs Pellequer et al. (2011); Plantier et al. (2012). **(B)** In detail, the GNN algorithms’ training process starts by extracting sub-graphs from the residue network obtained from pre-processed the FVIII-RIN. Next, the sub-graphs are used to train a Graph Attention Network (GAT) with four attention heads. After computing the attention scores, GAT utilizes a Multilayer Perceptron (MLP) to classify the graph nodes according to the severity of hemophilia A or the coagulation activity of the FVIII alanine mutants.

In previous studies, we created a residue interaction network (RIN) of the FVIIIa protein, where each residue was represented by a node, and two nodes were connected by an edge if the residues were close to each other in the 3D structure (Figure 1A). This representation of the FVIIIa protein helped us quantify the importance of each of its residues, and understand how perturbations (i.e., mutations), lead to the loss of its function (Yan et al., 2014).

In this study, to create a RIN, we used the FVIIIa structure predicted by AlphaFold2 (Jumper et al., 2021; Varadi et al., 2022), because it had a very good agreement with experimentally determined structures (Ngo et al., 2008; Shen et al., 2008; Smith et al., 2020), but in contrast to these models, the AlphaFold2 structure did not have large missing segments–an essential requirement to create a complete residue network.

We used the FVIIIa structure as input to RINerator (Doncheva et al., 2011). This program first adds hydrogen atoms to the structure, allowing it to identify non-covalent interactions between amino acids. Next, the non-covalent interactions are identified using a small probe (∼0.25 Å) rolled around the van der Waals surface of each residue, and a contact is defined if the probe touches two non-covalently bonded atoms (Word et al., 1999a; Word et al., 1999b). Finally, the interactions between residues are represented by edges, indicating that these residues are connected by a i) side-chain–side-chain, ii) side-chain–main-chain, iii) main-chain–main-chain hydrogen bond or non-covalent interaction between their atoms. In the FVIIIa RIN, the distance between the residues’ atoms was ∼5 Å (Supplementary Table S1 contains the complete network).

In mathematical terms, we modeled our data as a graph G=(V,ξ) , such that V is a set of residues and *ξ* is a set of edges that represents the connection between two nodes, i.e., there is a connection (*u*, *v*) ∈ *ξ* if two amino acids u,v∈V are in close proximity on the FVIII 3D structure. Here, the graph contains only undirected edges. Therefore, by using GNN, it is possible to train graph-based models (*f*) representing the connections from the protein structures, thus describing better the attributes and relationships according to our class labels (f:G→Y) (e.g., the severity of hemophilia).

Finally, we used the 3D structure of FVIIIa to calculate the relative surface exposure of each residue, to produce a large multiple sequence alignment of FVIIIa and obtain a conservation score of each of its residues (Methods). In practical terms, these measures quantify from a structural and evolutionary perspective the importance of each residue of FVIIIa (Figure 1).

Taken together, in addition to the centrality measures that can be derived from the graph itself, we aimed at ranking the importance of each FVIIIa residue from different perspectives. The FVIIIa RIN and these additional measures compose the base for input to the GNN algorithms.

### 2.2 Predicting hemophilia a severity with graph-based machine learning classifiers

**FIGURE 2:**
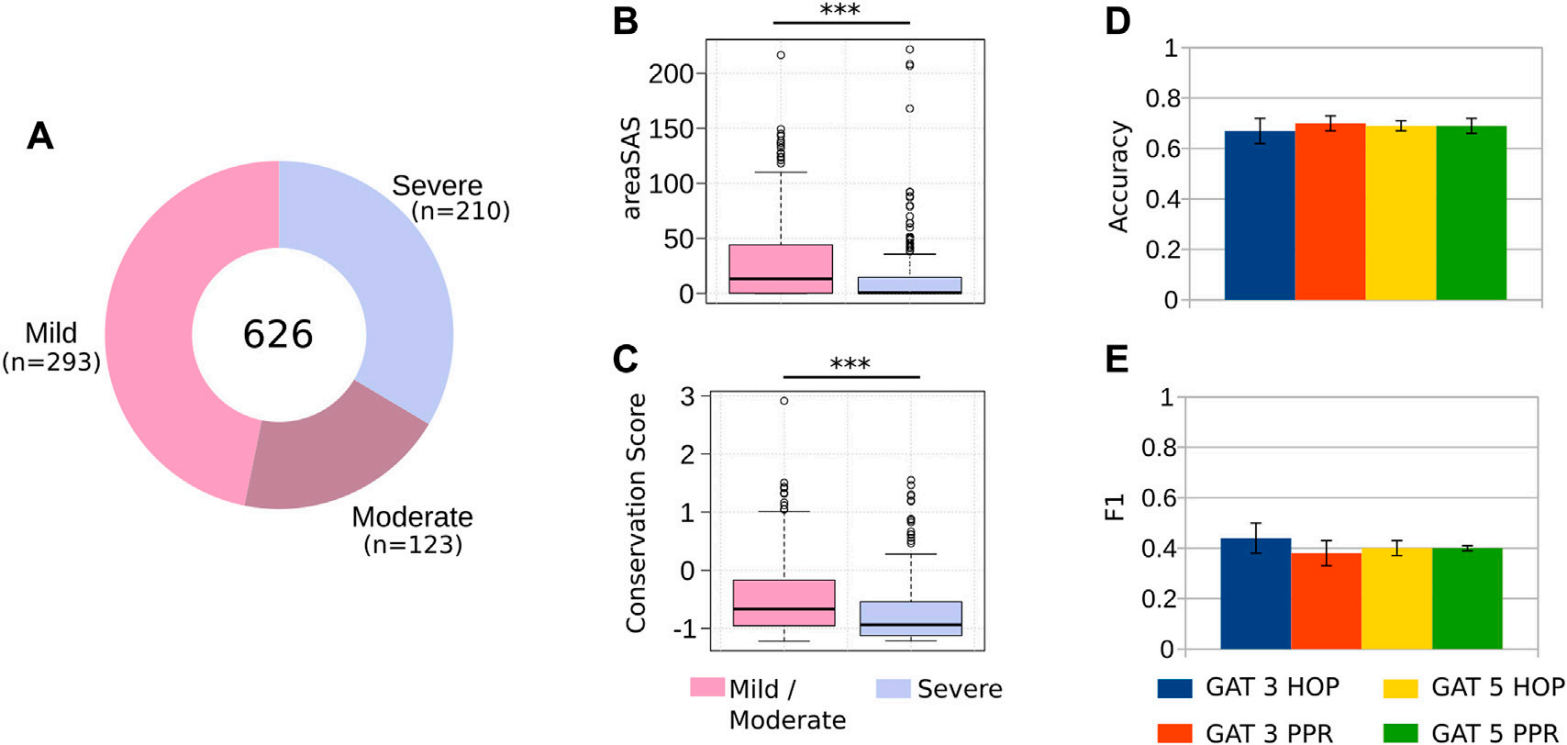
Predicting the severity of HA. **(A)** After careful data sanitation, our dataset had 626 unique cases of HA (Supplementary Table S2), caused by single-point, non-synonymous mutations. We merged the mild and moderate cases into a single class, reducing the problem to a 2-class classification. **(B–C)** Mutations at residues buried at the core of FVIII (i.e., low solvent accessible area), and conserved during evolution (i.e., low conservation score) result in severe HA, most likely due to the disruption the FVIII protein conformation. **(D–E)** Comparing different classifiers’ architectures, we obtained a classification accuracy of 0.69, and F1 value of 0.44, highlighting the difficulty associated to predicting the severity of HA from clinical data, but still useful to anticipate the effects of single-point mutations.

After generating the datasets that form the basis for GNN classifiers, we manually collected and pre-processed thousands of HA cases of patients harboring single-point non-synonymous mutations (Methods). After our stringent data sanitation, our dataset contained 626 HA cases (293 mild, 123 moderate and 210 severe), as well as the position and the amino acid substitution of each patient (Figure 2A). While patients with severe HA require prophylactic care that consists of intravenous injections 2-3 times per week, treatment for mild and moderate cases often require intravenous injections only when an injury occurs (Prezotti et al., 2022). For this reason, we grouped the mild/moderate cases into one class and severe into another (majority class ratio of 0.66). Mathematically, *χ* represents a dataset with the mutations harbored by hemophiliac patients with different severity levels Y . Each residue *x*_*i*_ ∈ *χ* contains a set of attributes $A=\left\{a_{1},a_{2},\ldots ,a_{n}\right\}$ representing the properties of them which was substituted in *x*_*i*_ (namely, the structural and evolutionary features of each amino acid).

Before using machine learning classifiers (ML) to predict the severity of HA, we assessed whether the structural and evolutionary properties of the FVIII residues could distinguish between severe and mild/moderate phenotypes. We found that the solvent accessible area and the conservation of residues are powerful discriminators of HA severity (Figure 2B), as we observed in a previous study with different clinical cases (Lopes et al., 2021a). These results indicate that mutations to the most conserved and buried residues of FVIII lead to severe hemophilia, while substitutions of the residues close to the protein surface are associated to mild or moderate phenotypes.

Next, we used structural and evolutionary measures in conjunction with the FVIII-RIN for the GNN-based classification. The predicting model used in our GNN-HemA framework was implemented on top of the SHADOW-GNN (Decoupled GNN on a shallow subgraph) (Zeng et al., 2021), which is considered the state-of-the-art for implementing different GNN models. With the Shadow-GNN, we created a experimental setup using Graph Attention Networks (GAT) (Veličković et al., 2017) to model the FVIII protein.

We have trained four GAT models combining different numbers of layers (3 and 5) and sub-graph extractors (L-HOP and PPR). In GAT, layers refer to the repeated application of a particular computation on the graph’s nodes, which is used to learn node representations (Methods).

To assess our results, we designed our experiments by using a 6-fold cross-validation strategy. We split our dataset into 6 folds due to the small amount of available data, i.e., by using more folds (e.g., 10 folds as usual in ML tasks), a larger number of nodes were available for training, but only a few would remain for the validation stage.

Using this training regimen and comparing the performance of the different GNN architectures, we found that the best model GAT with 3-PPR predicted the severity of HA with accuracy of 0.7 and F1 value of 0.44 (Figures 2D, E), indicating that the GNN models are able to find with modest performance the characteristics distinguishing severe and mild/moderate HA phenotypes (Figure 2C). Compared to existing methods that attempt to predict harmful effects of mutations (e.g., Polyphen-2 and Provean (Adzhubei et al., 2013; Choi and Chan, 2015), the GNN-Hema produced equivalent results in all cases (Supplementary Figure S1).

In summary, the best prediction of HA severity was obtained using the Shadow-GAT, an attention-based architecture for classifying nodes in graph-structured data (Veličković et al., 2017). By using a self-attention strategy, Shadow-GAT computes hidden representations of each node. This attention architecture has several desirable features (Veličković et al., 2017), including the fact that it is efficient and parallelizable, can handle nodes of varying degrees by assigning arbitrary weights to neighbors, and is suitable for inductive learning [i.e., the tasks where the model must generalize to new, unseen graphs (Veličković et al., 2017)].

### 2.3 Predicting *in vitro* activity

After using the GNN-Hema to predict the severity of HA in patients, we wanted to assess the feasibility of using the same framework to predict the effect of targeted alanine mutations. For this purpose, we used the coagulation activity and the antigen levels of 344 alanine mutations on the A2 and the C2 domains of the FVIII protein (Pellequer et al., 2011; Plantier et al., 2012). The A2 domain is the most important domain of this protein, as it has binding sites for FIXa and for FX [the members of the tenase complex (Lee et al., 2014)]. Moreover, by itself, the A2 domain is able to enhance the activity of FIXa [albeit with lower efficiency compared to the full-protein (Fay and Koshibu, 1998; Fay et al., 1999)]. The C2 domain exerts multiple activities, including interaction with the membrane of platelets and binding to the von Willebrand Factor (Inaba et al., 2022). Hence, anticipating the effects of mutations in these domains can enhance the understanding of vital FVIII functions.

**FIGURE 3:**
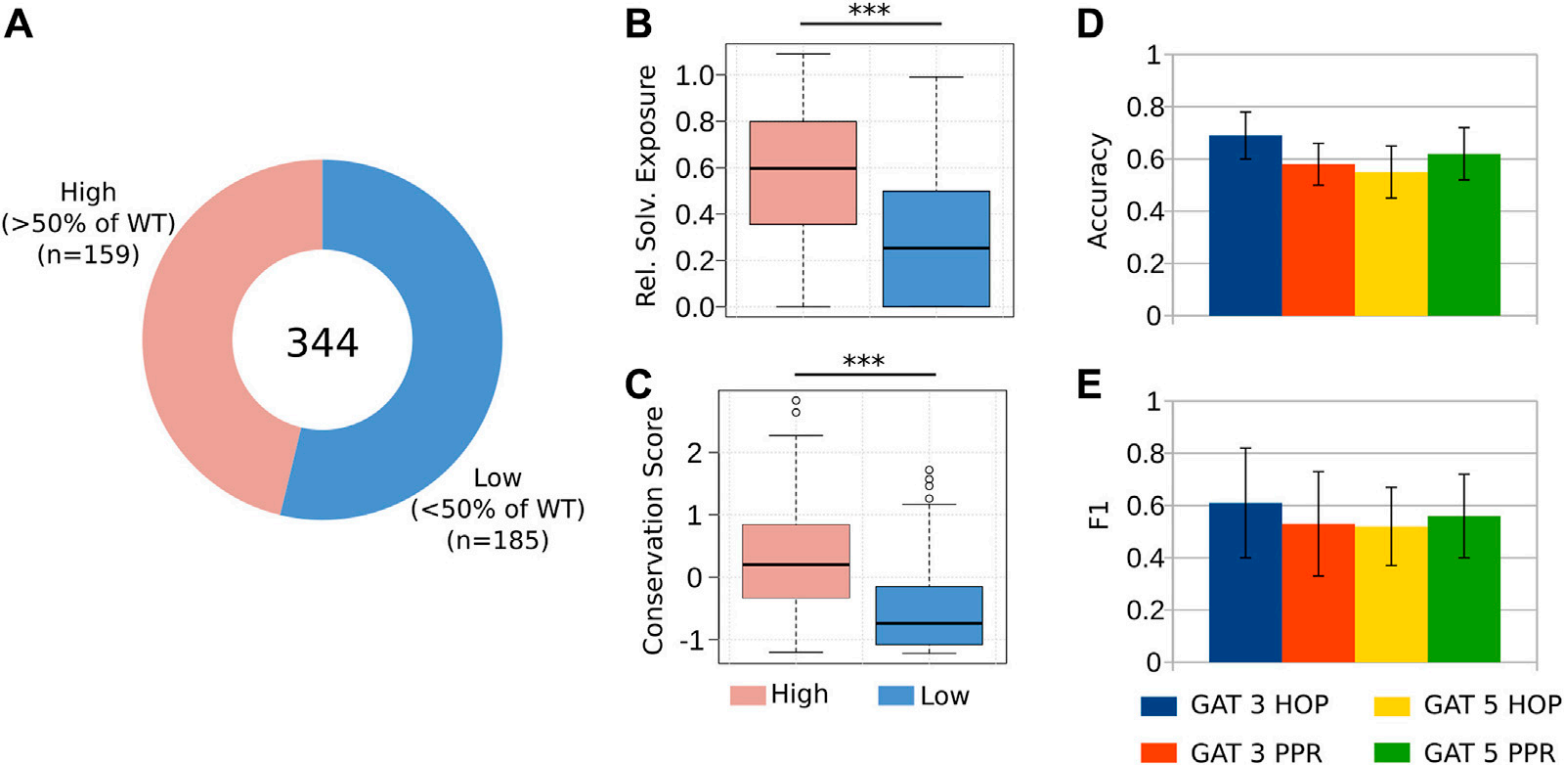
Predicting the reduction of coagulation activity in alanine mutants. **(A)** We considered 344 alanine mutations to the A2 and the C2 domains of FVIII. We divided these mutations into two groups, namely, those that retained at least 50% of the coagulation activity of the WT, and those below this threshold, measured by a chromogenic assay (Pellequer et al., 2011; Plantier et al., 2012) (Supplementary Table S3). **(B–C)** As it happens with clinical cases, the targeted mutations at the core hydrophobic residues and to those that are highly conserved, impair the co-factor activity of FVIII (Lopes et al., 2021b). **(D–E)** The GAT 3 HOP architecture presented the best predictive power, with an accuracy of 0.7 and F1 value of 0.61, indicating that this GNN model can be used to simulate *in silico* the effect of targeted alanine mutations to FVIII.

First, we divided our dataset into two-classes, namely, the mutations that retained a medium or high coagulation activity of FVIII, and the mutations that considerably disrupted its function (coagulation activity >50% and <50% of WT, respectively; Figure 3A; Supplementary Table S3). We verified that substitutions of residues buried at the core of FVIII and conserved during the course of evolution, often reduce dramatically the coagulation activity of the recombinant proteins (Lopes et al., 2021b; Lopes et al., 2022; Figures 3B, C). Next, we used this dataset as input to the GNN-Hema, together with the FVIIIa-RIN and the structural and evolutionary measures of all residues, and observed that the GNN could classify the alanine mutations in the A2 and the C2 domains with accuracy of 69% and F1 of 0.61 (Figures 3D, E). While these results do reach the threshold necessary for clinical diagnosis, in practice they help reduce the number of candidates designed to generate recombinant FVIII proteins.

**FIGURE 4:**
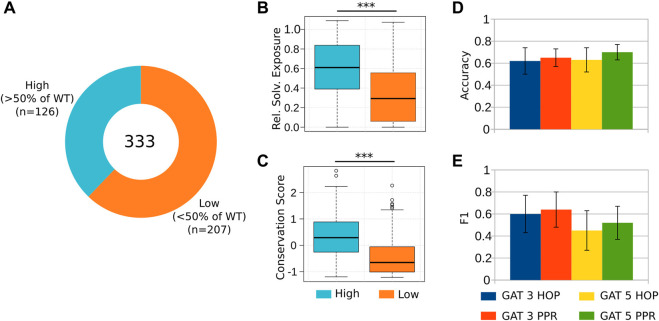
Predicting the reduction of coagulation activity in alanine mutants. **(A)** We considered 333 targeted alanine mutations to the A2 and C2 domains of FVIII. We divided these mutations into two groups, namely, those that retained at least 50% of the coagulation activity of the WT, and those below this threshold, measured by an ELISA (antigen) assay (Pellequer et al., 2011; Plantier et al., 2012) (Supplementary Table S4). **(B–C)** As expected, substitutions of the residues located at the core of these domains, as well as the most conserved ones, result in poor rescue of recombinant proteins by ELISA, suggesting that these mutations affected to a higher extent the correct folding and expression of FVIII. **(D–E)** The classification evaluation emphasizes GAT 5 PPR and GAT 3 PPR presented the best accuracy and F1 results, respectively.

Next, we aimed to predict the antigen levels of the 333 alanine mutant proteins (Plantier et al. (2012); Pellequer et al. (2011); Figure 4A; Supplementary Table S4). The antigen was measured by “sandwich” ELISA. This assay was used to evaluate the effectiveness of expressing and secreting FVIII mutant constructs. The ELISA assay is also known as an antigen assay because it measures both functional and nonfunctional FVIII proteins by measuring the amount of FVIII antigen (protein) that is immobilized on the ELISA plate. Once more, we observed that substitutions of the most buried and conserved residues of the A2 and C2 domains lead to a major reduction of the antigen rescue levels of the mutants [Lopes et al. (2021b; 2022)] (Figures 4B, C). We found that the GAT-5 PPR architecture successfully distinguished mutants that retained high antigen levels ( >50% of WT), against those that displayed low antigen levels ( <50% of WT; Figures 4D, E). These results indicate that the GNN-Hema reliably identify mutants with unusual conformation, and as the shape defines the function in the protein world, these predictions can be used to discard unpromising candidate molecules.

Together, these results indicate that using GNN-based classifiers is a viable approach to emulate *in silico*, the mulecular perturbations that could only be obtained by *in vitro* experiments.

## 3 Discussion

In this study, we established a computational pipeline to anticipate the effects of mutations in the FVIII protein. We found that representing its structure as an undirected graph, and using it as input for graph-based neural networks is viable to predict the severity of hemophilia A in patients harboring non-synonymous mutations. Moreover, the same classifiers were retrained to predict the loss-of-function of more than 300 targeted alanine mutations (Pellequer et al., 2011; Plantier et al., 2012), establishing an helpful resource for the rational design of recombinant therapeutic FVIII proteins.

The so-called protein residue networks are well-studied representations that enable researchers to ellucidate the underlying 3D biophysical and biological properties (Yan et al., 2014). For instance, there is a close relationship between the centrality of nodes in a network, and the level of disruption to the protein function caused by mutations [i.e., substitutions of the most central nodes lead to a complete loss-of-function (Amitai et al., 2004)]. Additionally, protein networks have helped to ellucidate the organization of amino acids into modules, maintaining the correct positioning of binding sites (del Sol et al., 2006). In our case, we leveraged on this knowledge to created residue networks specifically aimed at studying the effect of mutations related to hemophilia A (Lopes et al., 2021b), hemophilia B (Lopes et al., 2022), and to thrombosis (Lopes et al., 2023). While we obtained good results in those studies, we used only general-purpose ML algorithms suitable for tabular data.

Here, we introduced the use of GNNs–a more close representation that learn directly from a graph structure, without having to first calculate centrality measures and convert them to tabular data. In general, the execution of GNNs depends on encoder-decoder functions to represent the graph as node embeddings, which is processed by using Neural Message Passing (NMP). Each message-passing iteration performed during the training phase, new knowledge from node embeddings are updated according to information aggregated from their neighborhoods (Zeng et al., 2021). This approach displayed positive results when predicting new edges and node importance (Hamilton, 2020). However, the fundamental difference between these applications and the present study is the size of the datasets used.

While previous studies used graphs of millions of nodes and edges, our hemophilia datasets had only a few hundred cases–as is often the case when researching rare diseases. After comparing several GNN architectures and training regimens, we observed that it is possible to predict with reasonable certainty the effect of substitutions of the FVIIIa residues. This compares well with our previous studies (Lopes et al., 2021b), and surpassed existing alternatives (Adzhubei et al., 2013; Choi and Chan, 2015) (Supplementary Figure S1). As others also observed, predicting the effect of harmful or benign mutations is a difficult problem in the structural biology field (Broom et al., 2020), but there are high hopes placed on strategies based on deep-learning (Akdel et al., 2022).

In particular for the study of hemophilia, we are aware of the factors that hinder a more favorable prediction of mutation effects. First, there are known inconsistencies in the diagnosis of patients due to difficulties in standardizing reagents, discrepancies between one- and two-stages assays (Potgieter et al., 2015), and the reported diagnosis and what is observed in terms of bleeding frequencies (Inaba et al., 2022). Moreover, albeit the GNN models used here are the state-of-the-art algorithms (Zeng et al., 2021), they were not designed for small datasets; this requires its underlying architecture to be modified, varying the number of layers to properly extract implicit information from the FVIII proteins. Moreover, we have fine-tuned the hyperparameters to adjust the final model to our data, thus reaching the best performance in classifying the hemophilia severity (Methods). Yet, we are confident that with sequencing technology becoming widely available, and a vibrant community continuously improving GNN algorithms, the field is headed for accurate and personalized diagnostics.

In conclusion, the GNN-Hema is to our knowledge, the first application of graph-based classifiers to predict the effect of mutations to the FVIII protein–an application urgently required for diagnosis and for the generation of superior recombinant proteins. We implemented GNN-Hema as an open-source application, anticipating that the research community will extend and repurpose it to study other diseases.

## 4 Materials and methods

### 4.1 Creation of the RIN

We downloaded the FVIII structure generated by AlphaFold 2 (Jumper et al., 2021; Varadi et al., 2022), and removed the residues of the initial signal peptide, and the a1, a2 and B domain regions (residues −19 to −1, 336-372, 711-740, 741-1,689, respectively, in the legacy numbering system), because they had low modeling quality (pLDDT). Hence, our FVIIIa structure started at the residues Ala-Thr-Arg.

We used the Rosetta software suite release 280 and the ref2015 score function (Leaver-Fay et al., 2011) to find the most appropriate rotamer conformation of all residues, in a way to minimize the overall free-energy of the structure. We used the parameters -ignore_unrecognized_res -relax:constrain_relax_to_start_coords -relax:coord_constrain_sidechains -relax:ramp_constraints false -ex1 -ex2 -use_input_sc and generated 100 structures as output, from which we selected the one with the lowest energy score.

We used this structure as input to RINerator version 0.5.1 (Doncheva et al., 2011), which rely on the Probe and Reduce programs (Word et al., 1999a; Word et al., 1999b). In the first step, it adds hydrogen atoms to the structure, which is essential to identify non-covalent interactions between amino acids, and second, it identifies the non-covalent interactions using a small probe (approximately 0.25 Å), rolled around the van der Waals surface of each amino acid, and a contact is established if the probe is simultaneously in contact with two non-covalently bonded atoms.

We considered that two residues interacted if there was at least one edge between them, independent of the edge type. To analyze the FVIII-RIN, we used R version 3.6.3 (R Core Team, 2022) and the iGraph package, version 1.2.5 (Csardi and Nepusz, 2006). With the iGraph package, we used the function simplify to remove redundant edges and self-interactions. We visualized the networks using Cytoscape version 3.8.2 (Shannon et al., 2003).

### 4.2 Structural and evolutionary measures of FVIII

We used Chimera version 1.14 (Pettersen et al., 2004) to extract the solvent-excluded area (areaSES) and the solvent-accessible surface area (areaSAS), and to calculate the relative surface exposure of all amino acids from the customized FVIIIa structure. We divided the solvent-excluded area of the residue by the surface area of the same type of residue in a reference state; in our case, we used the reference values of the 20 standard amino acids in Gly-X-Gly tripeptides (Bendell et al., 2014). Moreover, we obtained the conservation score from the ConsurfDB webserver (Ben Chorin et al., 2020), using the FVIII protein structure as input for the search query.

### 4.3 Genetic data and mutations datasets

For the training and prediction of the severity of HA, we manually searched the EAHAD and the CHAMP FVIII mutation databases (McVey et al., 2020) (https://www.cdc.gov/ncbddd/hemophilia/champs.html; visited in 19 April 2022), and searched for single-point, non-synonymous mutations. We remove conflicting instances, such as those reported with multiple phenotypes at the same time (e.g., “Mild/Moderate”), or with mismatches between the residue position and the actual amino acid, as well as those that introduced a stop codon. Moreover, if there were multiple phenotypes reported for the mutations at the same position, we kept those that could be disambiguated by majority voting. Our final dataset had 626 instances (293 mild, 123 moderate, 210 severe). For the residue network used as input to the GNNs to predict the HA severity, we selected only the edge with the highest score between two residues, independent of the edge type (e.g., main-chain - main chain, or side-chain - side-chain). Next, we normalized the weights of all selected edges to the interval [0,1], and used the areaSAS and the conservation of each residue of the network in conjunction as input to the GNNs.

For the training and prediction of the coagulation activity and the antigen levels of the FVIII alanine mutants (Pellequer et al., 2011; Plantier et al., 2012). We divided the dataset into two classes ( >50% percent of WT, and <50% percent of WT). For the residue network used as input to the GNNs, we selected only the edge with the highest score between two residues, independent of the edge type (e.g., main-chain - main chain, or side-chain - side-chain), and normalized the weights of all selected edges to the interval [0,1], but reversed it, so that the most meaningful edges had a higher score. We used the relative surface exposure and the conservation of each residue of the network in conjunction as input to the GNNs.

### 4.4 The GNN architecture

As previously mentioned, the GNN models used to learn from the FVIII protein structure were trained using shadow-GNN (Decoupled GNN on a shallow subgraph) (Zeng et al., 2021) by using the steps summarized in Figure 1B.

The next step is responsible for extracting sub-graphs from the protein structure. The SHADOW implementation contains two extractors: i) *L*-HOP, which retrieves an entire or a random subset of the target node’s *L*-HOP neighbors; and ii) PPR, which uses the Personalized PageRank (PPR) algorithm to compute the scores of other nodes relative to the target node, then selects the top K nodes with the highest scores. In our experiments, we define the hyperparameter space for *L*-HOP extractor as Depth (*L* = 2), Budget (*b* = 20); and for PPR extractor as: Budget (*b* = 150), with thresholding (*epsilon* = 1*e* − 5).

In the subsequent phase, the outputs obtained from the extractors were utilized to optimize the parameters of our GNN. Specifically, in this study, we have trained a Graph Attention Network (GAT) architecture with four attention heads. In essence, attention heads compute the importance of different interactions (e.g., node-node), keeping the focus on the most relevant information in the graph. As output in our case, attention heads provide scores to weigh the contribution of nodes to the final representation of the graph.

Following the computation of the attention scores, GAT utilizes a Multilayer Perceptron (MLP) network to classify the nodes in the graph. In the present study, the MLP network is implemented with a hidden dimension of 256, a dropout rate of 0.35, random subset aggregation (drop edge) of 0.1, a learning rate of 1*e* − 3 and a batch size of 128. These specific hyperparameter settings were chosen based on the results obtained from our experimental evaluations, and were selected to optimize the performance of the GNN model. Next, we used the Relu activation function $\left(f\left(u\right)=\max\left\{u,0\right\}\right)$ to process a given MLP output *u* and provide the estimated target. Finally, it is worth mentioning that all hyperparameters were set in an experimental setup using the vanilla version of Shadow-GNN.

## 5 Statistical analysis

The validation metrics used to assess the GNN model were the accuracy and the F1, as usually considered in the literature (Veličković et al., 2017; Zeng et al., 2021). Both metrics were calculated from the contingency tables produced by the validation process, considering the number of True Positive (*TP*), True Negative (*TN*), False Postive (*FP*), and False Negative (*FN*). The accuracy was calculated using Eq. 1, in which *n* represents the total number of nodes used to validate the final models. The F1 measure was calculated using Eq. 2, such that Eqs 3, 4 compute the recall and precision measures, respectively.

$$acc=\frac{TP+TN}{n}$$ (1)

$$F1=\frac{2\cdot \left(Recall\cdot Precision\right)}{Recall+Precision}$$ (2)

$$Recall=\frac{TP}{TP+FN}$$ (3)

$$Precision=\frac{TP}{TP+FP}$$ (4)

F1 complements the accuracy and majority ratio by combining information from precision and recall measures. Precision estimates the fraction of correctly classified nodes among the ones classified as positive, while recall is the fraction of total positive nodes indeed classified as positive (Fernández et al., 2018).

The statistical tests were performed using the R statistical package version 4.2.2 (R Core Team, 2022).

## Data Availability

The original contributions presented in the study are included in the article/Supplementary Material, further inquiries can be directed to the corresponding author. The GNN-HemA source code and datasets developed in this study are available at https://github.com/LabIA-UFBA/GNN-HemA.
