# Supplementary figures and images for: A graph-based machine learning framework identifies critical properties of FVIII that lead to hemophilia A
Source: Front Bioinform. 2023 May 10;3:1152039. doi: 10.3389/fbinf.2023.1152039 (PMC10206133; doi:10.3389/fbinf.2023.1152039)

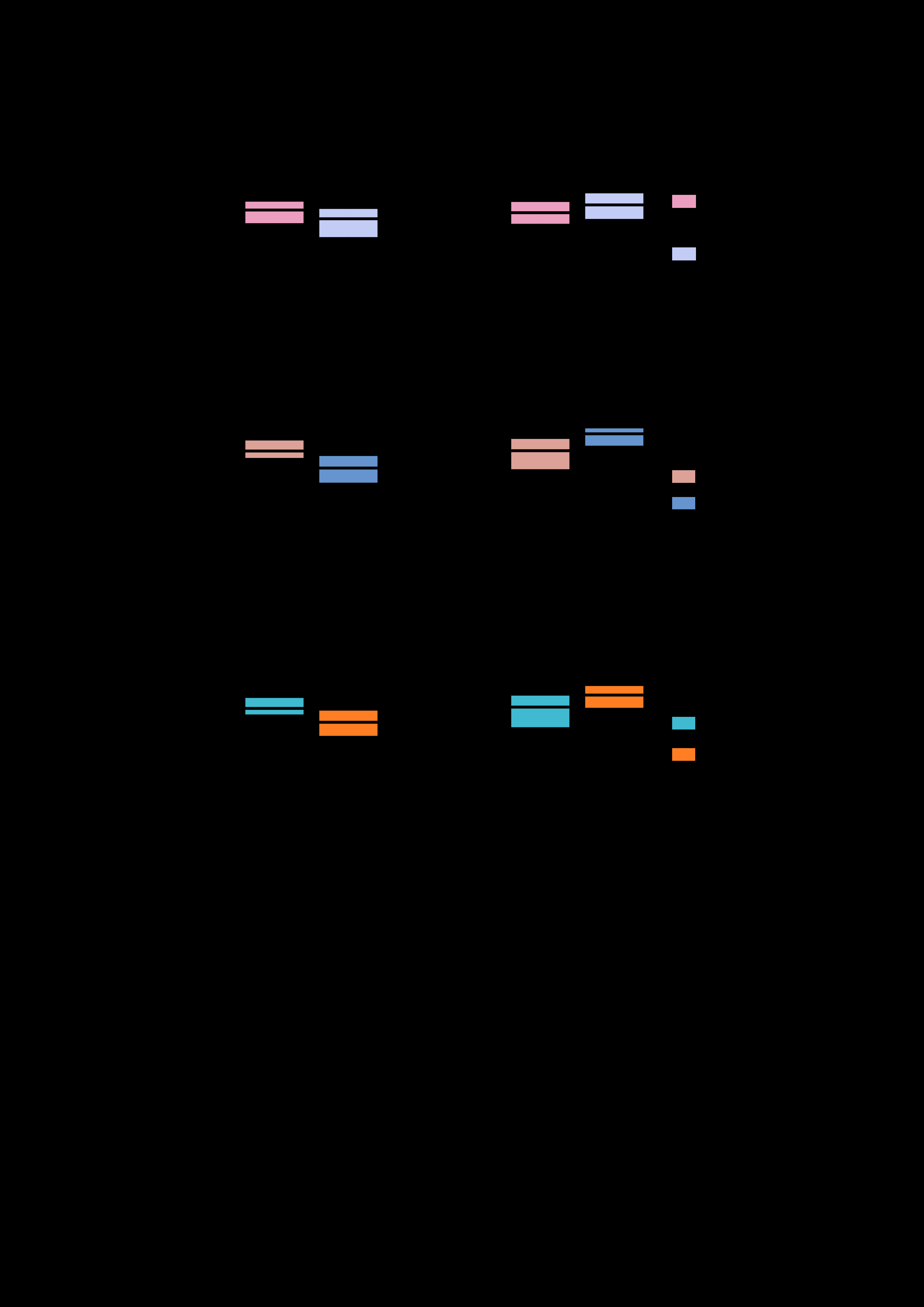

Supplement: Supplementary file 3 [file Image1.JPEG]

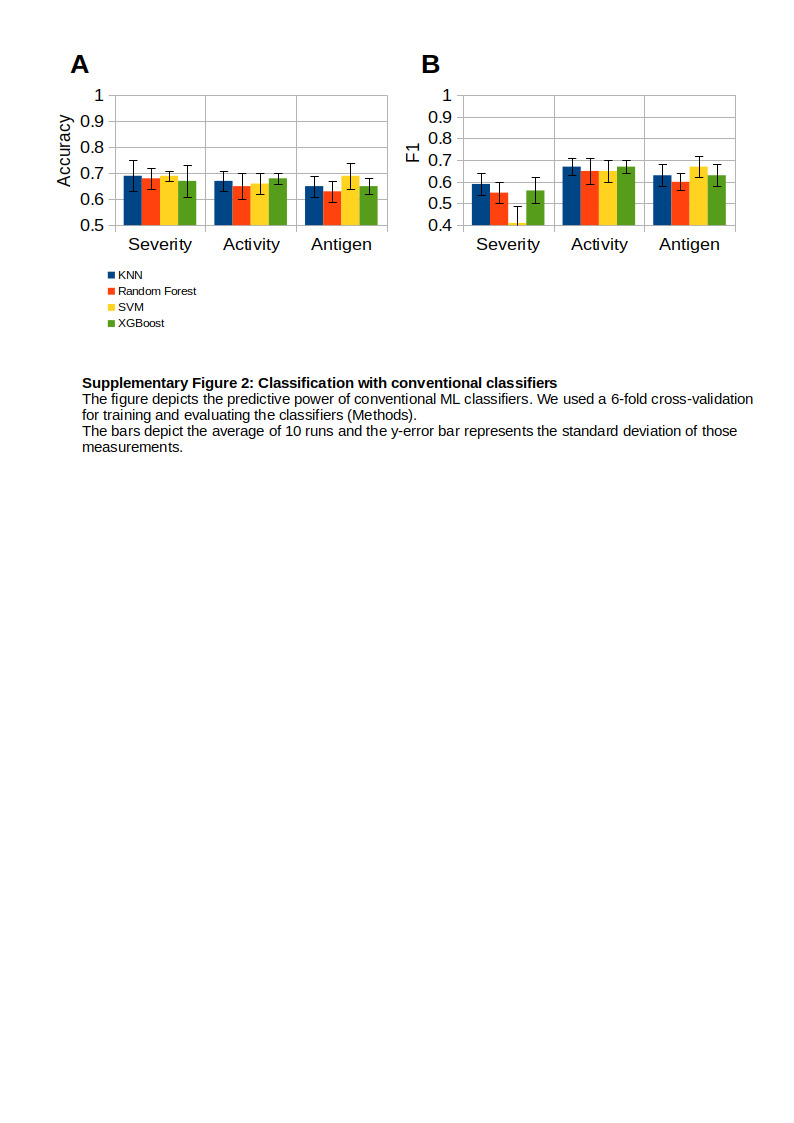

Supplement: Supplementary file 4 [file Image2.JPEG]
